# Identification of key genes and pathways related to cancer-associated fibroblasts in chemoresistance of ovarian cancer cells based on GEO and TCGA databases

**DOI:** 10.1186/s13048-022-01003-2

**Published:** 2022-06-23

**Authors:** Li Han, Xiaojuan Guo, Ruijuan Du, Kelei Guo, Pei Qi, Hua Bian

**Affiliations:** 1 Zhang Zhongjing School of Chinese Medicine, Nanyang Institute of Technology, Nanyang, 473004 PR China; 2 Henan Key Laboratory of Zhang Zhongjing Formulae and Herbs for Immunoregulation, Nanyang Institute of Technology, No. 80, Changjiang Road, Nanyang, 473004 Henan Province PR China; 3 Nanyang Traditional Chinese Medicine Hospital, Nanyang, 473007 PR China

**Keywords:** Ovarian cancer, Cancer-associated fibroblasts, Chemoresistance, MYC, EGFR, CCND1, P53 pathway, PI3K-Akt pathway, MAPK pathway

## Abstract

**Background:**

Studies have revealed the implications of cancer-associated fibroblasts (CAFs) in tumor progression, metastasis, and treatment resistance. Here, in silico analyses were performed to reveal the key genes and pathways by which CAFs affected chemoresistance in ovarian cancer.

**Methods:**

Candidate genes were obtained from the intersected differentially expressed genes in ovarian cancer, ovarian cancer chemoresistance, and ovarian CAF-related microarrays and chemoresistance-related genes from GeneCards databases. Kyoto Encyclopedia of Genes and Genomes enrichment analysis and Gene Set Enrichment Analysis were employed to identify the pathways engaged in ovarian cancer chemoresistance and ovarian CAF-related pathways. The top genes with high Degree in the protein-protein interaction network were intersected with the top genes enriched in the key pathways, followed by correlation analyses between key genes and chemotherapeutic response. The expression profiles of key genes were obtained from Human Protein Atlas database and TCGA-ovarian cancer data.

**Results:**

p53, cell cycle, PI3K-Akt, and MAPK pathways were the key pathways related to the implication of CAFs in ovarian cancer chemoresistance. 276 candidate genes differentially expressed in CAFs were associated with ovarian cancer chemoresistance. MYC, IGF1, HRAS, CCND1, AKT1, RAC1, KDR, FGF2, FAS, and EGFR were enriched in the key chemoresistance-related ways. Furthermore, MYC, EGFR, CCND1 exhibited close association with chemotherapeutic response to platinum and showed a high expression in ovarian cancer tissues and platinum-resistant ovarian cancer cells.

**Conclusion:**

The study suggests the key genes (MYC, EGFR, and CCND1) and pathways (p53, cell cycle, PI3K-Akt, and MAPK) responsible for the effect of CAFs on ovarian cancer chemoresistance.

## Introduction

Ovarian cancer is one of the most common malignant tumors in gynecology showing a high global incidence with over 3 millions of new cases and a high mortality with over 2 millions of deaths in 2020 [1]. Uncontrolled growth of ovarian cancer cells is the fifth leading cause of cancer death in women, as most ovarian cancer patients are diagnosed at the advanced stages of metastatic disease [2, 3]. Cytoreductive surgical treatment combined with platinum chemotherapy is considered to be optimal treatment of patients with advanced ovarian cancer [4]. However, most ovarian cancer patients who receive first-line platinum chemotherapy will ultimately develop platinum-resistant or platinum-refractory recurrent ovarian cancer [5].

Cancer-associated fibroblasts (CAFs) are activated fibroblasts with functional heterogeneity and constitute the central component of tumor microenvironment [6]. A subset of CAFs exert crucial role in epithelial-mesenchymal transition (EMT) and chemotherapy resistance through direct communicating with cancer cells or derived cytokines [7, 8]. In many cancers, CAFs promote tumor growth and are associated with low survival rates, which makes them potential therapeutic targets [9]. Accumulating evidences have shown the intimate connection between CAFs and ovarian cancer, for example, CAFs can promote tumor progression through interaction with tumor cells and enhance chemoresistance through multiple molecular mechanisms or pathways [10–12]. Furthermore, CAFs can activate the anti-apoptotic signal STAT3, thereby reducing cisplatin-induced apoptosis and promoting chemoresistance in ovarian cancer [13]. Previously, research has illustrated that CAFs secrete CCL5 in response to cisplatin treatment in ovarian cancer to increase resistance, which may be achieved through modulation of STAT3 and PI3K/Akt pathways [14]. In view of these findings, identification of genes and pathways mediated by CAFs in the chemoresistance of ovarian cancer cells potentially contributes to development of novel targets for reducing resistance in the treatment of ovarian cancer.

Bioinformatic analysis emerges as a promising tool for identifying potential therapeutic targets and prognostic markers of ovarian cancer and thus aids in the development of targeted therapies and individualized treatments [15]. The Cancer Genome Atlas (TCGA) involves a plenty of human tumors and profiles DNA, RNA, protein and epigenetic aberrations to give an integrated view of commonalities, differences and emergent events among tumor lineages [16]. Additionally, Gene Expression Omnibus (GEO) provides abundant gene expression profiles so as to offer a global integrated view of cellular regulation [17]. For instance, an integrated TCGA and GEO analysis has been comprehensively analyzed hundreds of dysregulated lncRNAs in ovarian cancer [18]. Herein, the study aimed to explore the key genes and pathways participated in the regulation of CAFs in ovarian cancer chemotherapy resistance based on GEO and TCGA databases.

## Materials and methods

### Microarray data for gene differential expression analysis

To explore the key genes and pathways by which CAFs could affect ovarian cancer chemotherapy resistance, DEGs were screened from the microarrays related to ovarian cancer and ovarian cancer chemoresistance searched in the GEO database. Ovarian cancer-related expression microarray GSE46169, ovarian cancer chemoresistance-related microarrays GSE15372 and GSE189717, and ovarian cancer CAF expression microarrays GSE126132 and GSE40595 were downloaded from the GEO database (https://www.ncbi.nlm.nih.gov/gds). GSE46169 contained 3 normal ovarian tissue samples and 27 ovarian cancer tissue samples. GSE15372 contained 5 samples of chemotherapy-sensitive cell lines and 5 samples of chemotherapy-resistant cell lines. GSE189717 contained 3 samples of chemotherapy-sensitive cell lines and 3 samples of chemotherapy-resistant cell lines. GSE126132 contained 10 CAFs samples and 24 epithelial cell samples. GSE40595 contained 8 normal ovarian stromal samples and 31 ovarian cancer stromal samples. Genes related to chemoresistance were searched through the GeneCards database (https://www.genecards.org/) and then ranked according to Relevance score, which was calculated considering the importance of different resources related to genes and disease. Additionally, DEGs in the ovarian CAF-related microarrays were screened and the genes related to chemoresistance were obtained from GeneCards database, and candidate genes were found in the intersection. In addition, UCSC Xena database (http://xena.ucsc.edu/) was employed to collect overall survival data of key genes by selecting GDC TCGA Ovarian Cancer (OV).

R language “limma” package (http://www.bioconductor.org/packages/release/bioc/html/limma.html) was applied to identify differentially expressed genes (DEGs) in GSE46169 and GSE15372, with |logFC| > 1 and *p* < 0.05 as the screening threshold. Besides, |logFC| > 0.6 and *p* < 0.05 were set as the threshold to identify DEGs in GSE40595. Heat map was plotted by utilizing “pheatmap” package (http://www.bioconductor.org/packages/release/bioc/html/pheatmap.html). The microarray GSE189717 was employed to verify the differential expression patterns of key genes in 3 chemotherapy-sensitive and chemotherapy-resistant cells. The results were analyzed statistically by *t* test with *p* < 0.05 considered to be significantly differentially expressed.

### Gene function enrichment analysis and gene set enrichment analysis (GSEA)

Gene function enrichment analysis was carried out to seek the crucial signaling pathways participated in chemoresistance in ovarian cancer. Kyoto Encyclopedia of Genes and Genomes (KEGG) enrichment analysis on the ovarian cancer chemoresistance-related key genes was conducted using R language “clusterProfiler” package (http://www.bioconductor.org/packages/release/bioc/html/clusterProfiler.html) with the threshold of *p* < 0.05 and bubble charts were plotted. Additionally, gene ontology (GO) and KEGG enrichment analyses of candidate genes (ovarian CAF-related) were performed, and bar charts and circle charts were plotted.

Then, we used ovarian CAF-related microarrays from the GEO database GSE126132 and GSE40595. To identify the pathways related to ovarian CAFs, GSEA was performed on the ovarian cancer CAF-related microarrays GSE126132 and GSE40595 using GSEA software (v4.0.0) and HALLMARK database, with |NES| > 1, NOM *p*-val < 0.05, FDR q-val < 0.25 as the threshold, and an Enrichment plot was drawn.

### Construction of PPI network and correlation analyses

The PPI network was constructed using the String database (https://string-db.org/cgi/input?sessionId=b92G0n5gdTKt&input_page_show_search=on) with species defined as human. The Degree value (the number of other genes interacted with the candidate gene in PPI network, namely the core Degree) was calculated, and the top 10 were selected for display according to the Degree value in order from large to small.

Correlation analysis of candidate genes was carried out using “Corrplot” in R software package (http://bioconductor.org/packages/3.13/bioc/html/maCorrPlot.html) to validate the results of PPI. Based on the results of GO and KEGG enrichment analyses, PPI network construction, and correlation analysis, the ovarian CAFs and chemoresistance-related candidate genes were screened.

### Identification of the intersected genes

DEGs from microarray GSE46169 and GSE15372 were intersected using jvenn tool (http://jvenn.toulouse.inra.fr/app/example.html). Additionally, candidate genes from GSE40595 were intersected with the genes screened from GeneCards database. Also, top genes enriched in key pathway enrichment analysis (KEGG-TOP10) were intersected with genes with higher degree value in protein-protein interaction (PPI-TOP10) to obtain the key genes.

### Kaplan-Meier (KM) survival analysis

To analyze the correlations between the key genes and the overall survival of ovarian cancer patients, R language “survival” package (http://bioconductor.org/packages/survival) was employed to perform KM survival analysis based on TCGA database with *p* value < 0.05 as the threshold. The KM-plotter website (http://kmplot.com/analysis/index.php?p=service&cancer=ovar) was utilized to analyze the correlations between candidate key factors and the survival of TCGA-ovarian cancer patients after platinum chemotherapy.

### Protein expression analysis

**Fig. 1:**
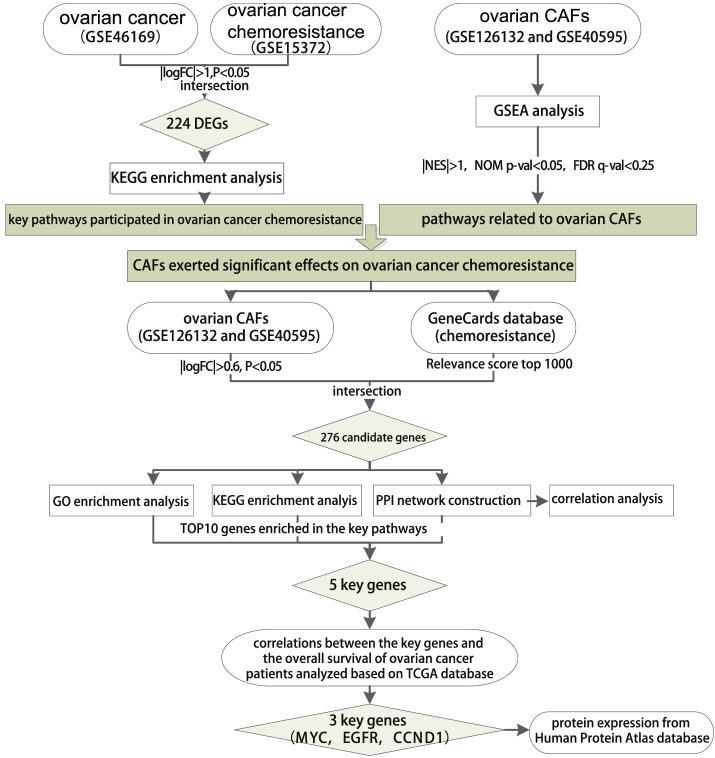
Schematic diagram of the key genes and pathways related to the function of CAFs in ovarian cancer chemoresistance

The key genes were further verified at the protein levels by immunohistochemistry (IHC) using Human Protein Atlas database (https://www.proteinatlas.org/) and corresponding staining images were downloaded. Finally, the key genes of CAFs affecting chemoresistance of ovarian cancer were determined (Fig. 1).

## Results

### p53, cell cycle, PI3K-Akt, and MAPK pathways are the key pathways related to ovarian cancer chemoresistance

**Fig. 2:**
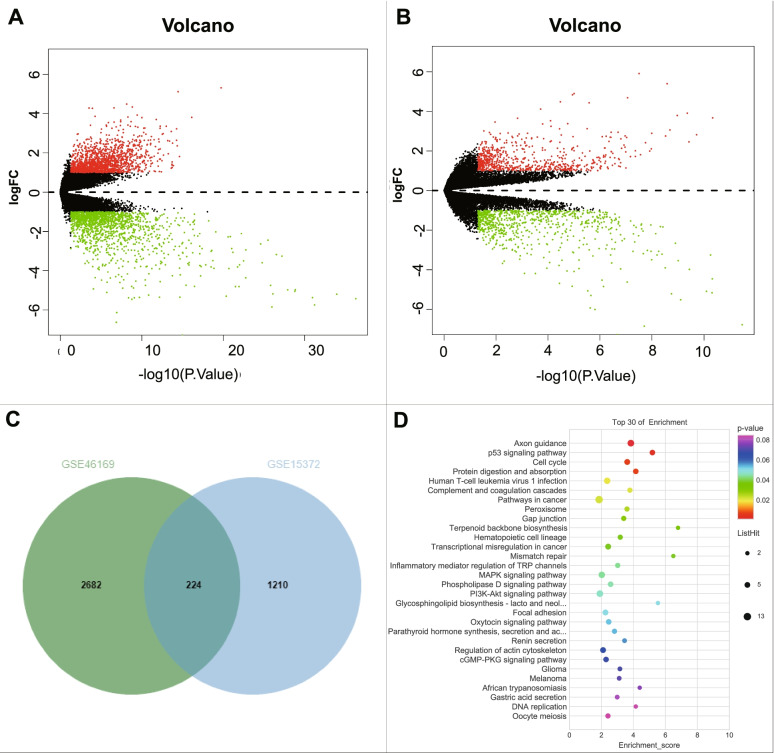
Screening of key genes for chemotherapy resistance in ovarian cancer and KEGG enrichment analysis. **A** Volcano plot of genes in ovarian cancer-related microarray GSE46169 (normal group, *n* = 3; tumor group, *n* = 27). **B** Volcano plots of genes in chemoresistance-related ovarian cancer cell-related microarray GSE15372 (cisplatin-sensitive group, *n* = 5; cisplatin-resistant group, *n* = 5). The red dots in Panels A and B represent significantly up-regulated genes, and the green dots indicate significantly down-regulated genes, black dots represent genes with insignificant expression differences. **C** The Venn diagram of the intersected genes in microarrays GSE46169 and GSE15372. **D** Bubble chart of KEGG enrichment analysis for 224 genes. The color of the dots indicates the *p* value, the size of the dots indicates the number of enriched targets, the x-axis indicates the number of enriched items, and the y-axis indicates the path name

Through the GEO database, we retrieved ovarian cancer-related microarray GSE46169 and ovarian cancer chemoresistance-related microarray GSE15372. The microarray GSE46169 contained a total of 2906 DEGs (Fig. 2A), and the microarray GSE15372 contained a total of 1433 DEGs (Fig. 2B). Finally, 224 genes related to chemoresistance in ovarian cancer were observed in the intersection (Fig. 2C).

According to the data from KEGG enrichment analysis on 224 genes, genes were found mainly enriched in p53 pathway (hsa04115), Cell cycle (hsa04110), Axon guidance (hsa04360), Protein digestion and absorption (hsa04974), PI3K-Akt pathway (hsa04151), and MAPK signaling pathway (Hsa04010) (Fig. 2D).

Recent studies have highlighted the important involvement of p53, PI3K-Akt, and p38 MAPK pathways in the chemoresistance of ovarian cancer cells [19–21]. Therefore, p53, cell cycle, PI3K-Akt, and MAPK pathways are considered as crucial pathways in influencing ovarian cancer chemoresistance.

### CAFs are enriched in the p53, mTORC1, and G2M checkpoint pathways

**Fig. 3:**
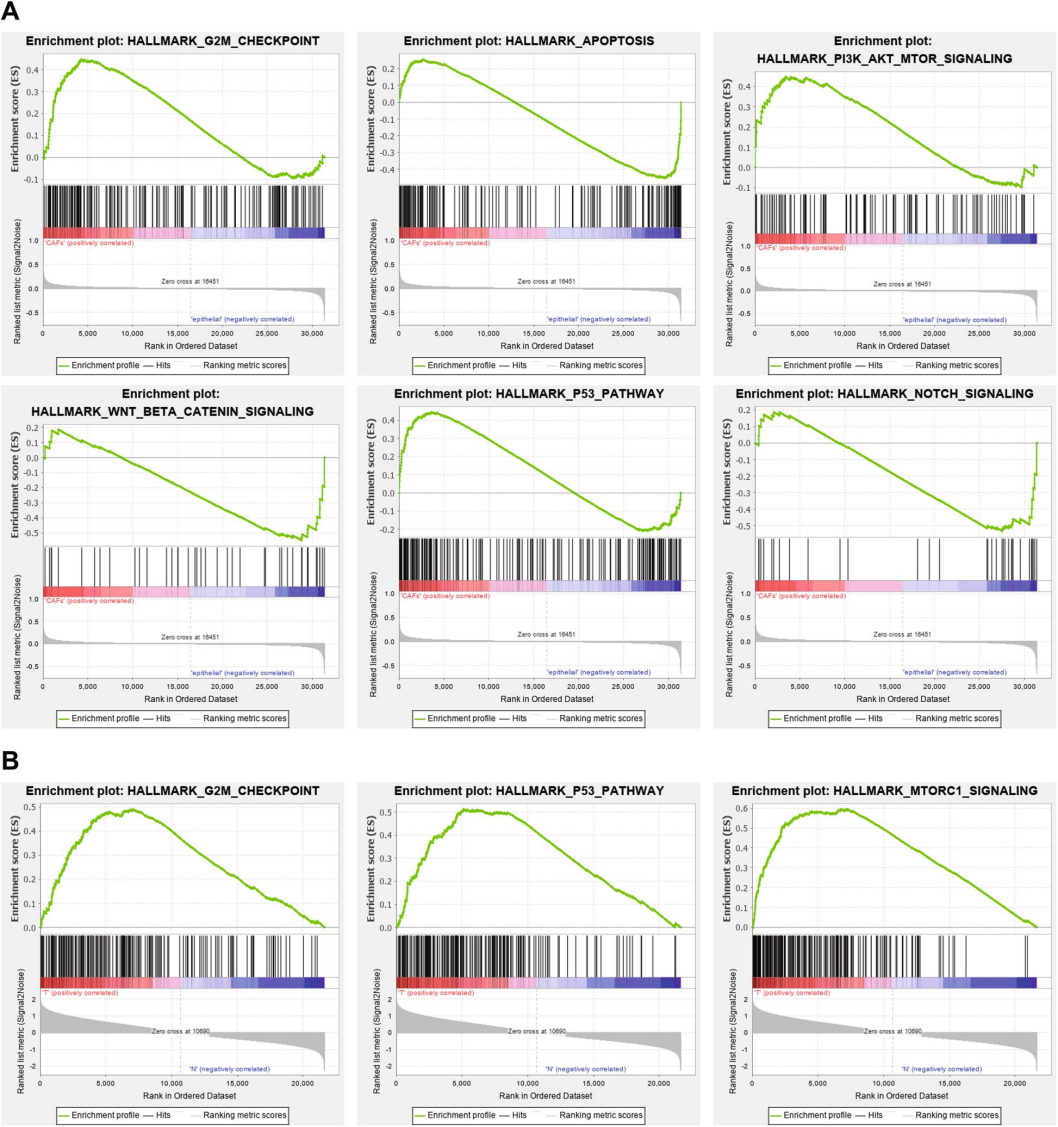
Enrichment of pathways related to CAFs. **A** Pathway enrichment of the genes in ovarian CAF-related microarray GSE126132 (CAFs, *n* = 10; epithelial cells, *n* = 24). **B** Pathway enrichment of the genes in the ovarian cancer stroma group in microarray GSE40595 (normal ovarian stroma group, namely N group, *n* = 8; ovarian cancer stroma group, namely T group, *n* = 31)

To explore specific mechanism responsible for the effect of CAFs on chemotherapy resistance in ovarian cancer, we retrieved ovarian CAF-related microarrays GSE126132 and GSE40595 in the GEO database and utilized GSEA to identify the pathways in which ovarian CAFs were primarily enriched. Based on microarray GSE126132, cells were divided into CAFs and epithelial groups, and the results showed that CAFs were mainly enriched in p53, PI3K-Akt, Notch, Wnt/β-catenin, G2M checkpoint, and apoptosis pathways (Fig. 3A). The microarray GSE40595 involved normal ovarian stroma and ovarian cancer stroma. As displayed in, Fig. 3B CAFs were enriched in the p53, mTORC1, and G2M checkpoint pathways.

### 276 CAF-related candidate genes exert an vital role in ovarian cancer chemoresistance

**Fig. 4:**
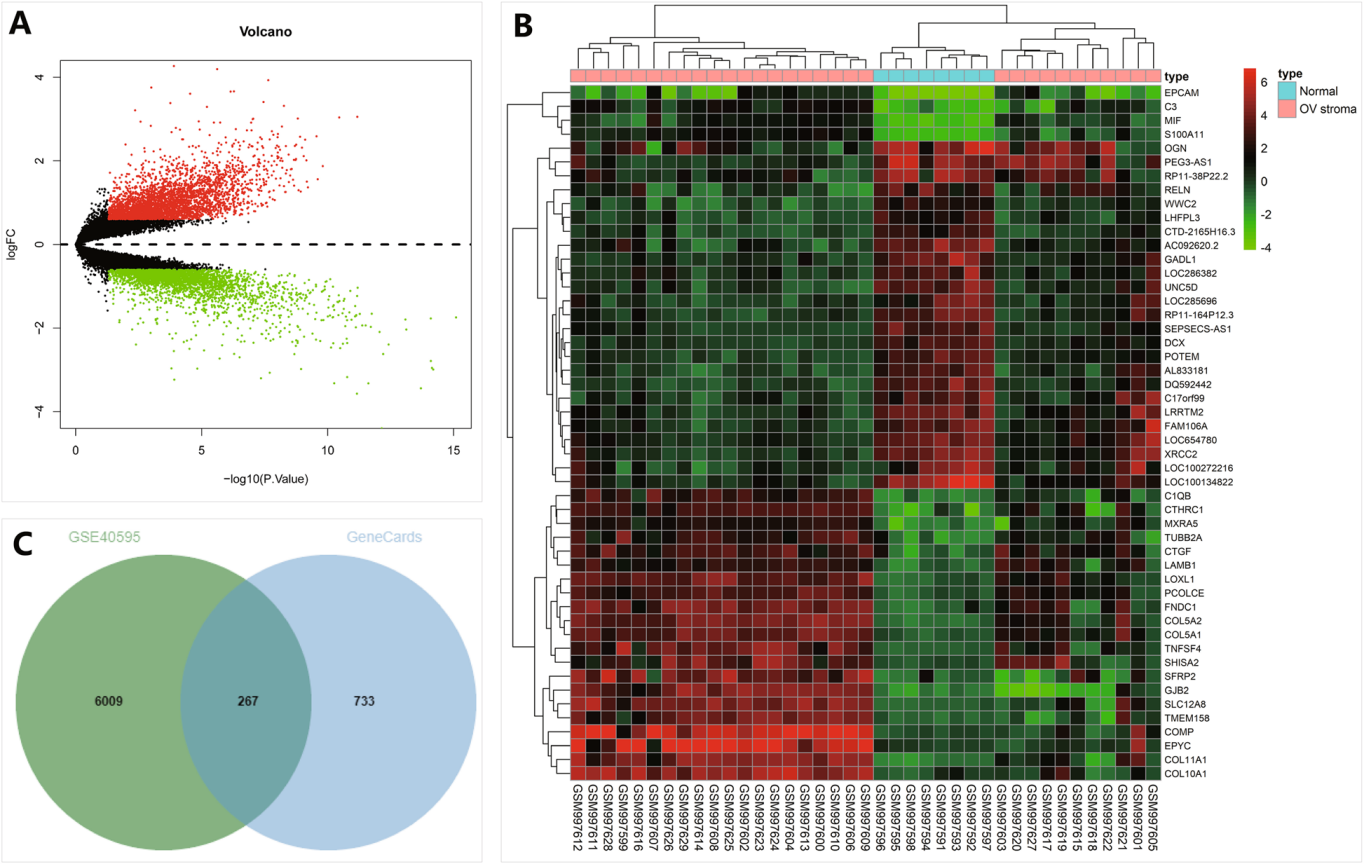
Candidate genes identified based on GEO and GeneCards databases. **A** The volcano plot of DEGs in the microarray GSE40595 (normal ovarian stroma group, namely N group, *n* = 8; ovarian cancer stroma group, namely T group, *n* = 31), the red dots represent significantly up-regulated genes, and the green dots indicate significantly down-regulated genes, black dots represent genes with insignificant differences in expression. **B** Heat map of TOP50 genes in microarray GSE40595. **C** The Venn diagram of top DEGs in the microarray GSE40595 and TOP50 genes from GeneCards database

To further explore the key genes that exert an vital role related to CAFs in ovarian cancer chemoresistance, differential analysis was performed on microarray GSE40595 associated with ovarian CAFs. DEGs were screened with the “limma” package in the R software, and heat map was plotted through the “pheatmap” package. GSE40595 contained 6276 DEGs. Volcano plots and heat map of TOP50 DEGs were shown in Fig. 4A-B. Meanwhile, we searched genes related to chemoresistance through the GeneCards database, and selected the top 1000 genes according to the Relevance score. Finally, 276 candidate genes were found in the intersection of the screened DEGs in GSE40595 and top genes from GeneCards database (Fig. 4C).

### Functional enrichment analysis of 276 candidate genes that may affect the resistance of ovarian cancer cells to chemotherapy

**Fig. 5:**
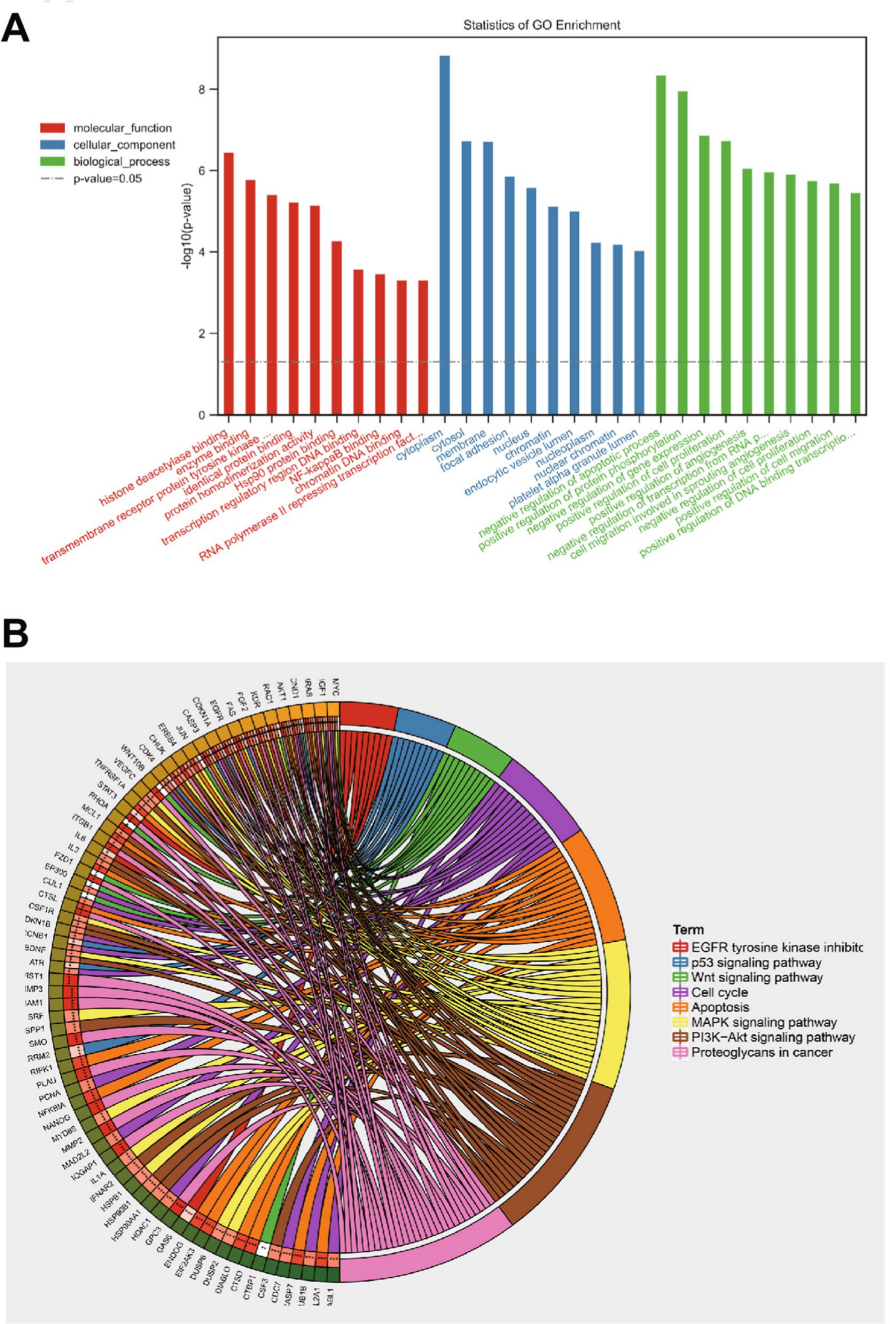
GO and KEGG enrichment analyses of 276 candidate genes. **A** Bar chart of GO enrichment analysis of candidate genes. The x-axis indicates the name of the pathway entry, and the y-axis represents the number of genes involved in the pathway. **B** The circle diagram of KEGG enrichment analysis of candidate genes, wherein the degree of gene enrichment decreases in the counterclockwise direction from MYC

Gene function enrichment analysis was performed on the aforementioned 267 candidate genes using the “clusterProfiler” package in the R software. GO enrichment analysis (*p* < 0.05) exhibited the significant effect of 256 molecular functions (MF) such as histone deacetylase binding (GO:0042826), enzyme binding (GO:0019899), and transmembrane receptor protein tyrosine kinase activity (GO:0004714). Also, 200 cellular components (CC) including Cytosol (GO:0005737), Cytosol (GO:0005829), and Membrane (GO:0016020) had significant effects. In addition, 944 biological processes (BP) such as negative regulation of apoptotic process (GO:0043066), positive regulation of protein phosphorylation (GO:0001934), and negative regulation of gene expression (GO:0010629) showed significant effects (Fig. 5A).

We here presented the results of KEGG enrichment analysis regarding the previously obtained key pathways related to CAF-affected chemoresistance (*p* < 0.05), and plotted the circle diagram to identify the key genes. It could be seen that MYC, IGF1, HRAS, CCND1, AKT1, RAC1, KDR, FGF2, FAS and EGFR are the top 10 genes in the circle diagram (Fig. 5B).

### Verification of the PPI of the top 10 key genes

**Fig. 6:**
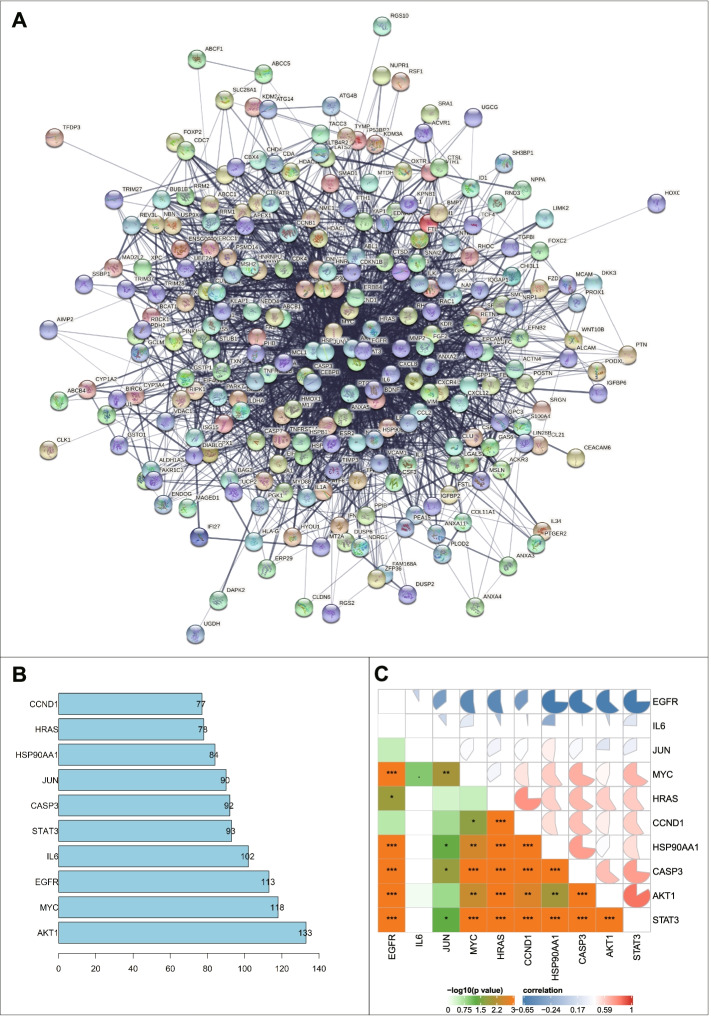
PPI and correlation analyses of candidate genes. **A** PPI network diagram of candidate genes. **B** Bar chart of the TOP10 genes with Degree value. **C** The correlation of TOP10 genes analyzed based on GSE40595 data

To further explore the PPI of these 267 candidate genes, we imported them into the String database and restricted the species to human. As shown in Fig. 6A, nodes represented proteins and edges represented correlations between proteins, involving 259 nodes and 2641 edges (PPI enrichment *p*-value < 1.0e-16). Degree (core degree) was the number of other genes interacted with the candidate gene in the PPI network. We sorted the top 10 according to the Degree value, namely AKT1, MYC, EGFR, IL6, STAT3, CASP3, JUN, HSP90AA1, HRAS, and CCND1 (Fig. 6B). Based on the data of 39 samples from the ovarian CAF-related microarray GSE40595, the “Corrplot” package in R software was utilized to conduct correlation analysis for the above 10 candidate genes, providing verification of PPI (Fig. 6C).

### MYC, EGFR, and CCND1 may affect the survival of ovarian cancer patients by mediating chemotherapy resistance-related pathways

**Fig. 7:**
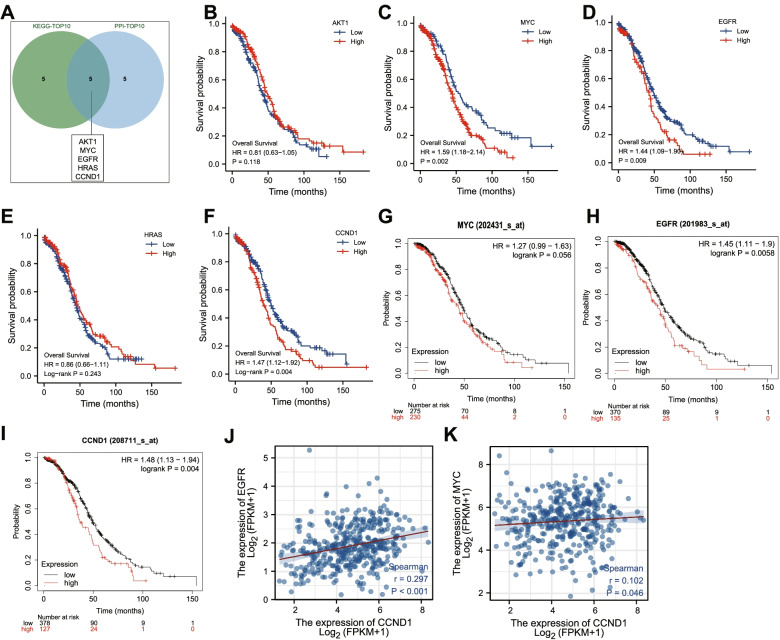
Correlation analyses of 5 key genes (AKT1, MYC, EGFR, HRAS, CCND1) with overall survival of patients with ovarian cancer. **A**, The Venn diagram of the intersection of KEGG-TOP10 genes and PPI-TOP10 genes. **B**-**F** Correlation analyses of AKT1 (**B**), MYC (**C**), EGFR (**D**), HRAS (**E**), and CCND1 (**F**) with the overall survival of ovarian cancer patients. **G**-**I** Correlation analyses of MYC (**G**), EGFR (H), and CCND1 (I) with the overall survival of ovarian cancer patients after platinum chemotherapy. **J** and **K** Correlation analyses of EGFR and CCND1 (**J**) expression as well as MYC and CCND1 (**K**) expression in TCGA-ovarian cancer data

Based on the ranks of genes in the enrichment analysis of the key pathways and the Degree value in PPI, top 10 genes were selected from both of which and 5 key genes (AKT1, MYC, EGFR, HRAS, CCND1) were obtained by taking the intersection (Fig. 7A). GDC TCGA Ovarian Cancer (OV) was selected in UCSC Xena database and the influence of the above 5 key gene expression on the overall survival of 758 ovarian cancer patients was analyzed. The results showed that MYC, EGFR and CCND1 were significantly correlated with overall survival, while AKT1 and HRAS were not correlated with overall survival (Fig. 7B-F).

Further, we selected the survival data of TCGA-ovarian cancer patients after platinum chemotherapy for KM survival analysis, and the results exhibited that the expression patterns of MYC, EGFR, and CCND1 were highly correlated with the survival of TCGA-ovarian cancer patients after platinum chemotherapy (Fig. 7G-I). Correlation analysis was performed based on the expression of MYC, EGFR, and CCND1 in the TCGA-ovarian cancer dataset. It was observed that CCND1 expression was significantly positively correlated with EGFR and MYC (Fig. 7J-K). Therefore, it was speculated that MYC, EGFR, and CCND1 might affect the survival of ovarian cancer patients by mediating chemoresistance-related pathways.

### Protein expression patterns of the key genes (MYC, EGFR, and CCND1) in ovarian cancer samples

**Fig. 8:**
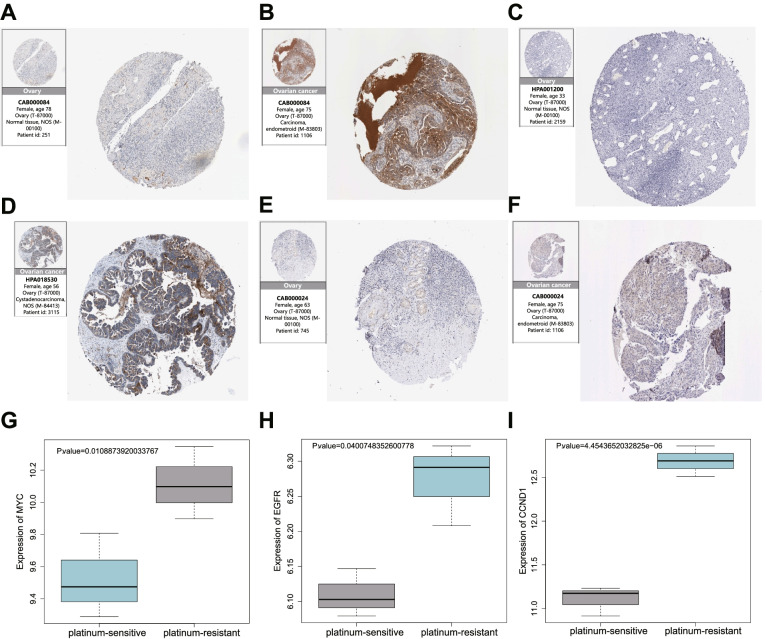
The expression of 3 key genes in ovarian cancer tissues and high-grade serous ovarian cancer cells. **A** and **B,** IHC results of MYC in normal ovarian tissues (**A**) and cancer tissues (**B**) based on Human Protein Atlas data. **C** and **D**, IHC results of EGFR in normal ovarian tissues (**C**) and cancer tissues (**D**) based on Human Protein Atlas data. **E** and **F**, IHC results of CCND1 in normal ovarian tissues (**E**) and cancer tissues (**F**) based on Human Protein Atlas data. **G**-**I** Expression plots of MYC (**G**), EGFR (**H**), and CCND1 (**I**) in the platinum-sensitive (*n* = 3) and platinum-resistant (*n* = 3) high-grade serous ovarian cancer cell samples based on GEO microarray (GSE189717)

The expression profiles of MYC, EGFR, and CCND1 were further verified at the protein level through IHC using the Human Protein Atlas database. MYC, EGFR, and CCND1 expressed at higher protein levels in ovarian cancer patients than in normal ovarian tissues (Fig. 8A-F). To further validate the relevance of MYC, EGFR, and CCND1 to platinum chemotherapy response, we analyzed their differential expression between platinum-sensitive and platinum-resistant high-grade serous ovarian cancer cells based on GEO microarray (GSE189717). Significantly high levels of MYC, EGFR, and CCND1 were detected in platinum-resistant high-grade serous ovarian cancer cell lines (Fig. 8G-I). Therefore, we speculated that MYC, EGFR, and CCND1 might enhance resistance to platinum chemotherapy in ovarian cancer.

## Discussion

Although approximately 75% of ovarian cancer patients initially respond to platinum/paclitaxel-based chemotherapy, most patients relapse and develop chemotherapy resistance, leading to treatment failure and more than 90% of deaths [22]. Drugs that target the components of tumor microenvironment have emerged as promising therapeutics for this deadly disease [23]. The effect of chemotherapy can be hampered by tumor epithelium, mainly by cellular autonomic mechanisms. Recently, tumor microenvironment is also shown to be an inducer responsible for chemotherapy resistance. CAFs constitute the most abundant cellular components in the tumor microenvironment [24] that can develop resistance to cancer cells, contribute to the initiation, progression and metastasis of cancer and confer resistance of cancer cells to drugs [25, 26]. For instance, CAFs are reported to be a contributor of chemotherapy resistance in colorectal cancer [27]. However, the specific mechanism underlying the action of CAFs in chemoresistance remains to be explored. This study mainly employed in silico analyses based on GEO and TCGA databases to reveal the key genes and pathways for CAFs affecting chemotherapy resistance in ovarian cancer. Consequently, this study unraveled MYC, EGFR, and CCND1 to be the key genes and p53, cell cycle, PI3K-Akt, and MAPK pathways the key pathways for the regulatory effect of chemoresistance in ovarian cancer.

At the beginning of this study, the key genes and pathways related to the role of CAFs in affecting chemotherapy resistance in ovarian cancer were screened using bioinformatics analysis. Bioinformatics is employed to obtain common DEGs in different gene expression profiles downloaded from GEO database so as identify molecular markers related to the prognosis of ovarian cancer [28]. After KEGG enrichment analysis, we found that CAFs exerted effects on chemotherapy resistance of ovarian cancer, and the key pathways involved which contained p53 pathway, cell cycle pathway, PI3K-Akt pathway and MAPK pathway. In line with our findings, CAFs promote ovarian cancer chemoresistance by modulating endothelial adhesion protein LPP [10]. CAFs are also strongly linked to the EMT and resistance of ovarian cancer cells to chemotherapy (cisplatin) [29]. Furthermore, several studies have addressed the involvement of the key pathways (p53, PI3K-Akt, and MAPK) in the chemoresistance of ovarian cancer cells. For instance, p53-mediated apoptotic pathway activation contribute to reducing chemoresistance of ovarian cancer cells [19]. Inactivation of PI3K/Akt/mTOR pathway can reverse EMT and inhibit cancer stem cell markers to overcome ovarian cancer chemoresistance [20]. Also, p38 MAPK pathway underlies the promotion of CUEDC2 in cisplatin resistance of ovarian cancer cells [21].

Our study revealed that 276 candidate genes were differentially expressed in CAFs and associated with ovarian cancer chemoresistance, and among which the top 10 genes MYC, IGF1, HRAS, CCND1, AKT1, RAC1, and KDR, FGF2, FAS, and EGFR were enriched in key pathways. Combined with results of PPI analyses and TCGA database, MYC, EGFR, and CCND1 were determined to be the key genes correlated with the chemoresistance affected by CAFs in ovarian cancer. MYC is enriched in MAPK, ErbB and p53 pathways, and MYC is significantly elevated in hepatocellular carcinoma [30]. CCND1 is up-regulated in human epithelial ovarian cancer tissues and cells, and its abnormal expression is related to the malignancy of epithelial ovarian cancer [31]. Clinical data on many ovarian cancer patients, including patient age, cancer site, stage and subtype, patient survival, and ovarian cancer gene transcription profiles, are publicly available from the TCGA dataset, which is applied to identify biomarkers related to disease progression and mortality [32]. Our study revealed significant correlations of MYC, EGFR, and CCND1 with overall survival based on TCGA data. A recent study has identified that MYC, EGFR, and CCND1 can be amplified on extrachromosomal DNA (ecDNA) in different tumor cell lines [33], which may provide novel insight into identification of the mechanism of ovarian cancer progression. Additionally, we observed that protein expression of MYC, EGFR, and CCND1 was notably increased in cancer tissues of ovarian cancer patients based on data from Human Protein Atlas, suggesting their important functional significance in ovarian cancer. A close correlation of MYC with chemoresistance can be found in colorectal, gastric, and ovarian cancers [34–36]. EGFR may play anti-autophagic role to affect tumor progression and chemoresistance by mediating Beclin 1 phosphorylation [37]. It has been recently proposed that EGFR motivates the chemoresistance of epithelial ovarian cancer cells via GFR/MEKK pathways [38]. Although the correlation of CCND1 to ovarian cancer chemoresistance is rarely mentioned, CCND1 is well-known as a cell cycle regulatory protein that participates in the regulation of cell proliferation and apoptosis in tumors [39]. A prior study has additionally revealed that cisplatin can downregulate CCND1 in human ovarian cancer epithelial cells which shares an association with delayed cell proliferation and boosted apoptosis [40]. GEO-based analysis of microarray GSE189717 showed ectopic expression of MYC, EGFR, and CCND1 in platinum-resistant high-grade serous ovarian cancer cell lines. Given these findings, we speculated whether MYC, EGFR, and CCND1 can be linked to the poor outcomes of ovarian cancer patients following chemotherapy. This was testified using TCGA-ovarian cancer data after platinum chemotherapy. Results exhibited positive correlations of MYC, EGFR, and CCND1 with overall survival of ovarian cancer patients after chemotherapy. Hence, targeting these genes might hold promise as anti-chemoresistance strategies for ovarian cancer patients. However, more experiments are needed for validation in the future before clinical translation, especially the possibility of CAF-based target therapies overcoming the chemoresistance of ovarian cancer.

## Conclusion

**Fig. 9:**
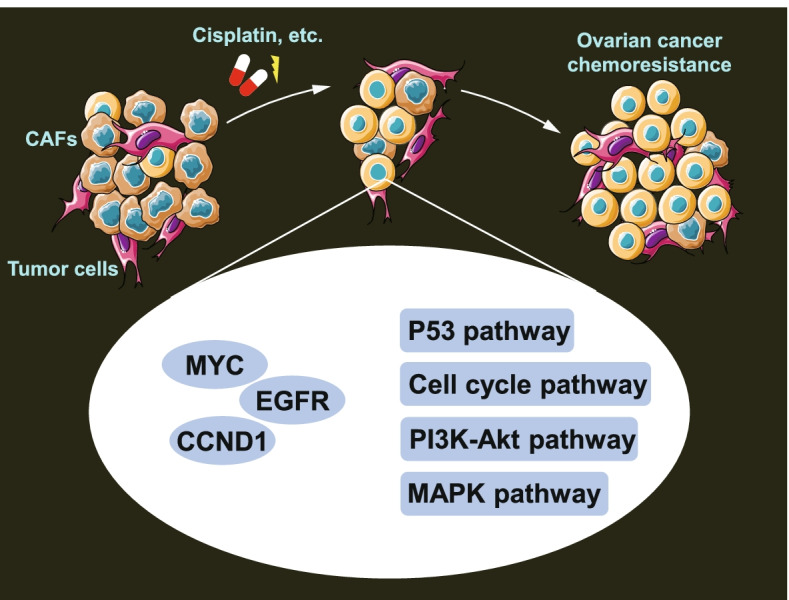
Schematic molecular mechanisms of key genes and key pathways that involve the role of CAFs in affecting chemoresistance in ovarian cancer

To sum up, our data suggest that MYC, EGFR, and CCND1 may be key genes by which CAFs act in ovarian cancer chemoresistance, and the p53 pathway, cell cycle pathway, PI3K-Akt pathway, and MAPK pathway may be the key pathways (Fig. 9). This study provides a new molecular theoretical basis for revealing the possible mechanism mediated by CAFs in affecting ovarian cancer chemotherapy resistance and offers new insight for the development of targets in CAF-based treatment for ovarian cancer patients.

## Data Availability

The datasets generated and/or analysed during the current study are available from the corresponding author on reasonable request.

## Abbreviations

CAFs: Cancer-associated fibroblasts
DEGs: Differentially expressed genes
GSEA: Gene Set Enrichment Analysis
PPI: Protein-protein interaction
EMT: Epithelial-mesenchymal transition
TCGA: The Cancer Genome Atlas
GEO: Gene Expression Omnibus
OV: Ovarian Cancer
GO: Gene ontology
GSEA: Gene Set Enrichment Analysis
IHC: Immunohistochemistry
ecDNA: Extrachromosomal DNA
